# Di­chlorido­(η^6^-*p*-cymene)[tris­(4-meth­oxy­phen­yl)phosphane]ruthenium(II)

**DOI:** 10.1107/S2414314621012591

**Published:** 2021-12-02

**Authors:** Wade L. Davis, Alfred Muller

**Affiliations:** a Department of Chemical Sciences, University of Johannesburg (APK Campus), PO Box 524, Auckland Park, Johannesburg, 2006, South Africa; Sunway University, Malaysia

**Keywords:** crystal structure, ruthenium, *p*-cymene, organometallic

## Abstract

The title compound, [RuCl_2_(C_10_H_14_)(C_21_H_21_O_3_P)], crystallizes with two complex mol­ecules in the asymmetric unit. The Ru^II^ atom has a classical three-legged piano-stool environment being coordinated by a cymene ligand [Ru—centroid = 1.707 (2)/1.704 (2) Å], a tris­(4-meth­oxy­phen­yl)phosphane ligand [Ru—P = 2.3629 (15)/2.3665 (15) Å] and two chloride atoms with the Ru—Cl bonds adopting two distinct values of 2.4068 (16)/2.4167 (16) and 2.4016 (15)/2.4244 (16) Å. The effective cone and solid angles for the phosphane ligands were calculated to be 149.5/150.2° and 25.3/25.6°, respectively. In the crystal, weak C—H⋯Cl/O/π inter­actions are observed. The crystal was refined as a two-component twin.

## Abstract 2

The title compound, [RuCl_2_(C_10_H_14_)(C_21_H_21_O_3_P)] crystallizes with two independent complex mol­ecules in the asymmetric unit. In the crystal, weak C—H⋯Cl/O/π inter­actions are observed.

## Graphical abstract

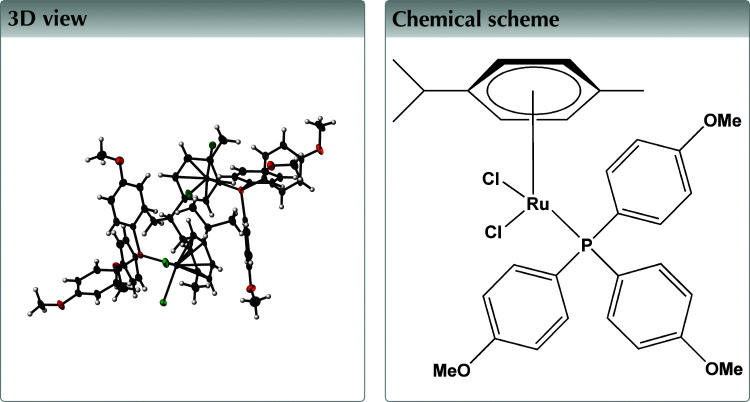

## Structure description

The activity of the half-sandwich Ru^II^–arene complexes is well known in the catalytic transfer hydrogenation of carbonyl compounds (Chen *et al.*, 2002 ▸; Crochet *et al.*, 2003 ▸; Aydemir *et al.*, 2011 ▸; Wang *et al.*, 2011 ▸). Reported here is the η^6^-cymene–Ru complex containing the phosphane, P(C_6_H_4_OMe-*p*)_3_, as part of ongoing structural investigations into these type of complexes.

**Figure 1:**
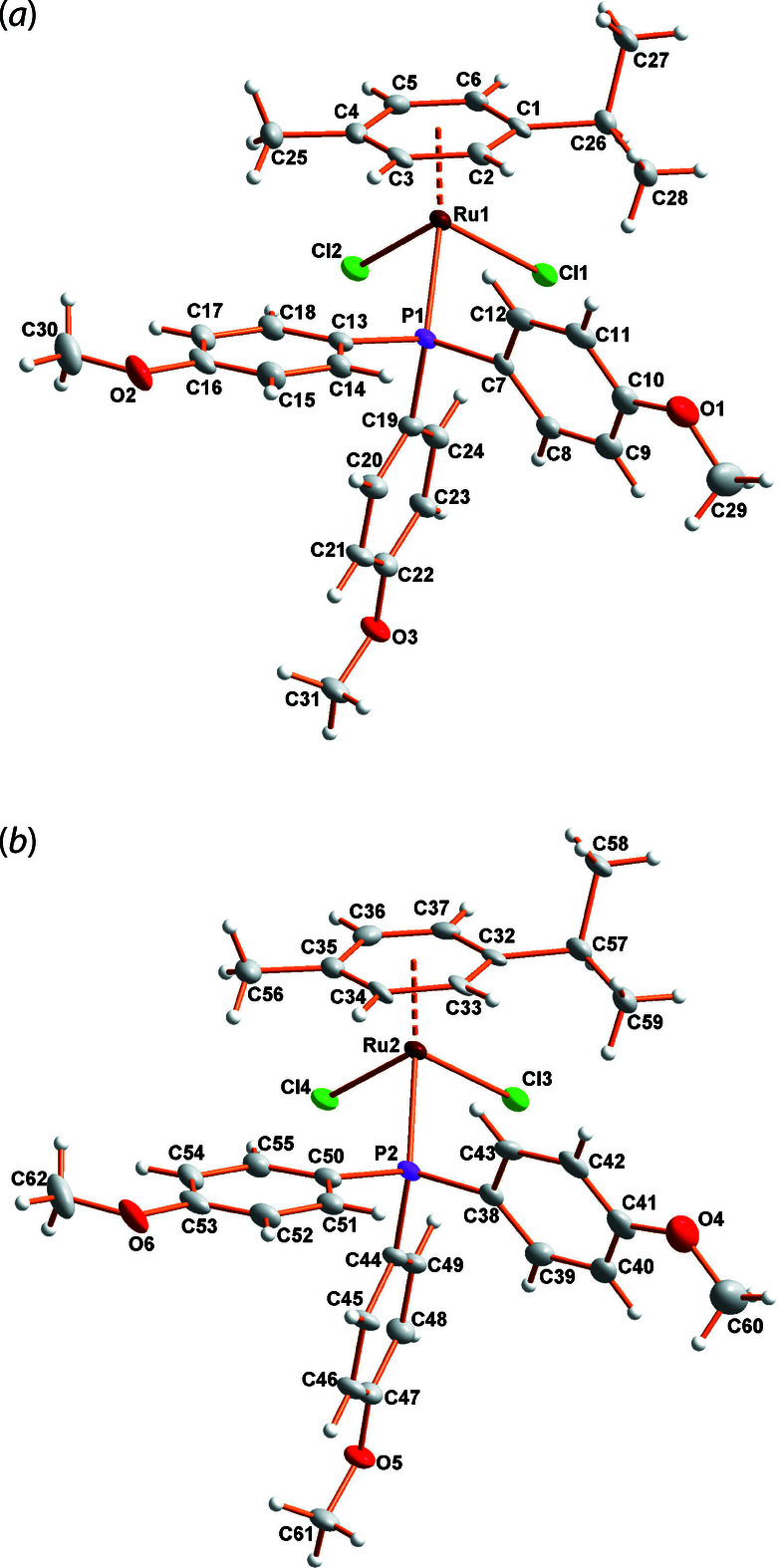
(*a*) and (*b*): Views of the title complex showing the atom-numbering scheme for the two independent mol­ecules in the asymmetric unit and 50% probability displacement ellipsoids. Mol­ecules were rotated independently to obtain the best view for each.

**Figure 2:**
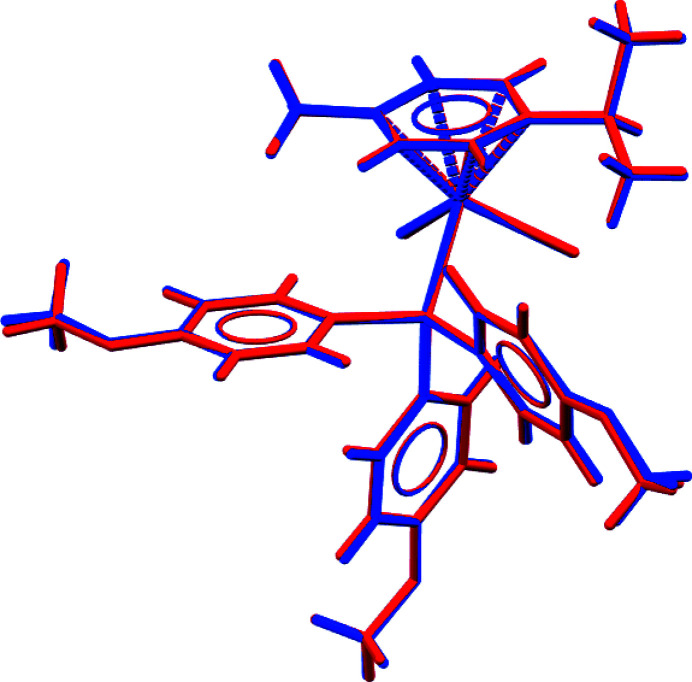
An overlay diagram showing the conformational similarity between the two mol­ecules in the asymmetric unit (r.m.s.d. = 0.0525 Å).

The title compound crystallizes in the triclinic space group *P* 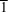 (*Z* = 4), with its two unique mol­ecules adopting a distorted pseudo-octa­hedral arrangement, revealing the typical three-legged piano-stool geometry. The coordination sphere of the ruthenium is occupied by a cymene, a tris­(4-meth­oxy­phen­yl)phosphane and two chloride atoms (see Fig. 1 ▸). The distances between Ru and the centroid of the π-bonded η^6^-cymene ligand are 1.707 (2) and 1.704 (2) Å for the two independent mol­ecules; the mean Ru—C bond lengths are 2.217 (6) and 2.214 (6) Å. The coordination of the remaining ligands to the Ru atom shows a slight deviation from the typical octa­hedral geometry with Cl—Ru—Cl = 88.47 (6) and 88.77 (6)°, respectively; Cl—Ru—P = 86.50 (5)/88.03 (5) and 86.05 (5)/88.21 (6)°. The Ru—P bond lengths are 2.3629 (15) and 2.3665 (15) Å, while the Ru—Cl bonds adopt two distinct values of 2.4068 (16)/2.4167 (16) and 2.4016 (15)/2.4244 (16) Å for Ru1 and Ru2, respectively. The above bond lengths are within normal ranges as data extracted from the Cambridge Structural Database (Allen, 2002 ▸) for (η^6^-ar­yl)RuCl_2_(P*R*_3_) systems from 429 hits, containing 535 usable Ru—Cl observations, show a mean value of 2.412 (12) Å in a range from 2.378 to 2.459 Å. The same group of structures show for the Ru—P distance a mean value of 2.34 (3) Å in a range from 2.235 to 2.434 Å. The geometries of the two independent mol­ecules are virtually identical, as seen from a superimposed fit with an r.m.s. deviation of 0.0525 Å (Macrae *et al.*, 2020 ▸; Weng, Motherwell, Allen *et al.*, 2008 ▸; Weng, Motherwell & Cole, 2008 ▸) (see Fig. 2 ▸).

**Figure 3:**
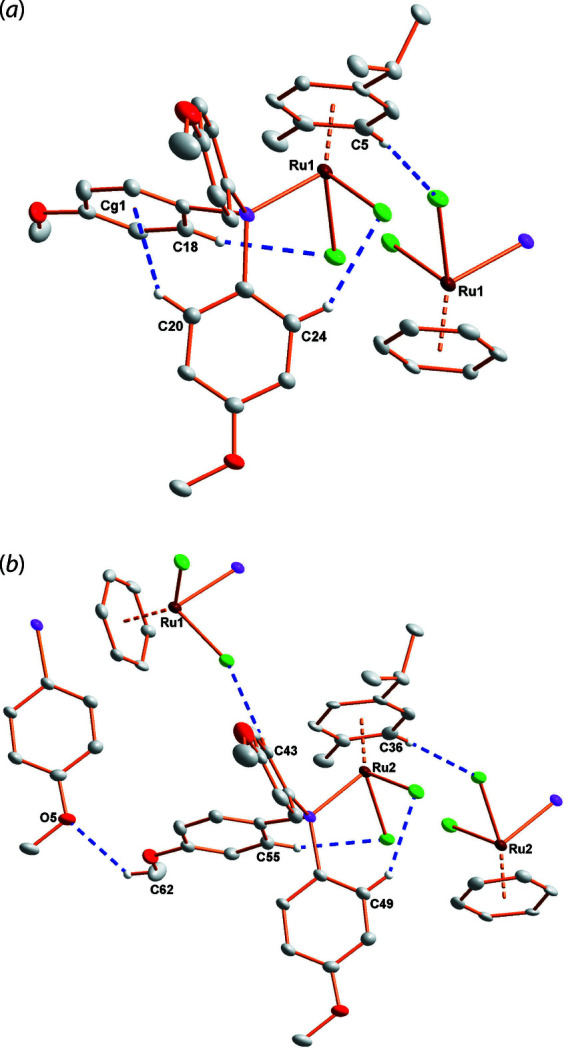
(*a*) and (*b*): Partial packing diagrams showing the C—H⋯Cl/O/π inter­actions (indicated by blue dashed lines). H atoms not involved in inter­actions are omitted for clarity.

To describe the steric demand of phosphane ligands, we have implemented the two most widely used models, *i.e.* the solid angle (a percentage projection of the ligand onto a sphere; Immirzi & Musco, 1977 ▸) and the crystallographic cone angle (an adaptation from the Tolman cone angle model; Tolman, 1977 ▸), where the orientation of the substituents are taken from crystallographic data instead of a CPK model, and the Ru—P bond length adjusted to 2.28 Å to normalize any influence this variation may have on the cone size (Müller & Mingos, 1995 ▸) to calculate an effective cone angle (Otto, 2001 ▸). The effective cone angle values obtained with this method for the two independent mol­ecules in the asymmetric unit are 149.5 and 150.2° compared to the Tolman cone angle of 145.0° obtained from the QALE website (Fernandez *et al.*, 2003 ▸). The solid angles, utilizing *SOLID-G* (Guzei & Wendt, 2004 ▸) were calculated as 25.35 and 25.61°. It is inter­esting to note that despite these similar geometric values, the phosphane ligands of these two independent mol­ecules show a marked variation in their orientations of substituents as the P1-phosphane has a C—H⋯π inter­action between two of its substituents, whereas the P2-phosphane does not show this feature. The rest of the crystal displays an array of weak C–H⋯Cl/O inter­actions (see Fig. 3 ▸, Table 1 ▸ for a summary of inter­actions).

## Synthesis and crystallization

A solution of P(C_6_H_4_OMe-*p*)_3_ (62.7 mg, 0.178 mmol) in CH_2_Cl_2_ (10 ml) was added to a stirred orange solution of [Ru(*p*-cymene)Cl_2_]_2_ (50 mg, 0.081 mmol) under Ar in the same solvent (15 ml) and stirred at r.t. for 24 h. The resulting red reaction mixture was filtered, the filtrate concentrated under reduced pressure to *ca* 5 ml. Cold diethyl ether (10 ml) was carefully added and the solvent left to slowly evaporate whereby a sample of [RuCl_2_(C_10_H_14_)(C_21_H_21_O_3_P)] suitable for single-crystal X-ray diffraction was obtained as orange crystals.

Analytical data: ^31^P{^1^H} NMR (CDCl_3_, 161.99 MHz): δ (p.p.m.) 21.39 (*s*). ^1^H NMR (CDCl_3_, 400 MHz): δ (p.p.m.) 1.11 (*d*, 6H, 2 × CH_3_ of isoprop­yl); 1.84 (*s*, 3H, CH_3_ of cymene); 2.87 (*m*, 1H, CH of isoprop­yl); 3.78 (*s*, 9H, 3 × CH_3_ of OMe); 4.93 (*d*, 2H, Ar—H of cymene); 5.20 (*d*, 2H, Ar—H of cymene); 6.85 (*dd*, 6H, Ar—H of C_6_H_4_OMe-*p*); 7.69 (*t*, 6H, Ar—H of C_6_H_4_OMe-*p*).

## Refinement

Crystal data, data collection and structure refinement details are summarized in Table 2 ▸. The deepest residual electron-density hole (−1.94 e Å^−3^) is located at 0.59 Å from Ru1 and the highest peak (3.95 e Å^−3^) 0.90 Å from Ru1. Initial refinement of data indicated a two-component twin with a 180° rotation about the [100] reciprocal direction. Refinement with the appropriate twin law yields a batch scaling factor of 0.18.

## Supplementary Material

Crystal structure: contains datablock(s) global, I. DOI: 10.1107/S2414314621012591/tk4072sup1.cif

Structure factors: contains datablock(s) I. DOI: 10.1107/S2414314621012591/tk4072Isup2.hkl

CCDC reference: 2124507

Additional supporting information:  crystallographic information; 3D view; checkCIF report

## Figures and Tables

**Table 1:** Hydrogen-bond geometry (Å, °) *Cg*1 is the centroid of the C1–C6 ring.

*D*—H⋯*A*	*D*—H	H⋯*A*	*D*⋯*A*	*D*—H⋯*A*
C5—H5⋯Cl2^i^	0.95	2.62	3.566 (7)	171
C36—H36⋯Cl4^ii^	0.95	2.6	3.506 (7)	159
C43—H43⋯Cl1^iii^	0.95	2.78	3.619 (7)	147
C62—H62*B*⋯O5^iii^	0.98	2.58	3.362 (9)	136
C18—H18⋯Cl2	0.95	2.8	3.643 (7)	149
C24—H24⋯Cl1	0.95	2.62	3.427 (6)	143
C49—H49⋯Cl3	0.95	2.61	3.416 (6)	143
C55—H55⋯Cl4	0.95	2.71	3.562 (6)	149
C20—H20⋯*Cg*1	0.95	2.95	3.614 (7)	128

Symmetry codes: (i) 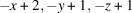 ; (ii) 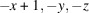 ; (iii) 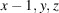 .

**Table 2:** Experimental details

Crystal data	
Chemical formula	[RuCl_2_(C_10_H_14_)(C_21_H_21_O_3_P)]
*M*_r_	658.53
Crystal system, space group	Triclinic, *P* 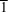
Temperature (K)	100
*a*, *b*, *c* (Å)	12.4069 (17), 14.0221 (19), 16.934 (2)
α, β, γ (°)	91.459 (3), 91.205 (3), 90.613 (3)
*V* (Å^3^)	2944.2 (7)
*Z*	4
Radiation type	Mo *K*α
μ (mm^−1^)	0.80
Crystal size (mm)	0.58 × 0.28 × 0.21

Data collection	
Diffractometer	Bruker APEX DUO 4K-CCD
Absorption correction	Multi-scan *SADABS* (Bruker, 2008 ▸)
*T*_min_, *T*_max_	0.654, 0.850
No. of measured, independent and observed [*I* > 2σ(*I*)] reflections	88599, 14838, 13368
*R*_int_	0.053
(sin θ/λ)_max_ (Å^−1^)	0.674

Refinement	
*R*[*F* ^2^ > 2σ(*F* ^2^)], *wR*(*F* ^2^), *S*	0.070, 0.181, 1.09
No. of reflections	14838
No. of parameters	698
H-atom treatment	H-atom parameters constrained
Δρ_max_, Δρ_min_ (e Å^−3^)	3.95, −1.95

Computer programs: *APEX2* (Bruker, 2011 ▸), *SAINT* and *XPREP* (Bruker, 2008 ▸), *SIR97* (Altomare *et al.*, 1999 ▸), *SHELXL97* (Sheldrick, 2008 ▸), *DIAMOND* (Brandenburg & Putz, 2005 ▸), *publCIF* (Westrip, 2010 ▸) and *WinGX* (Farrugia, 2012 ▸).

## full crystallographic data

### Crystal data

**Table:**

[RuCl_2_(C_10_H_14_)(C_21_H_21_O_3_P)]	*Z* = 4
*M**_r_* = 658.53	*F*(000) = 1352
Triclinic, *P*1	*D*_x_ = 1.486 Mg m^−^^3^
Hall symbol: -P 1	Mo *K*α radiation, λ = 0.71073 Å
*a* = 12.4069 (17) Å	Cell parameters from 9270 reflections
*b* = 14.0221 (19) Å	θ = 2.6–28.5°
*c* = 16.934 (2) Å	µ = 0.80 mm^−^^1^
α = 91.459 (3)°	*T* = 100 K
β = 91.205 (3)°	Block, orange
γ = 90.613 (3)°	0.58 × 0.28 × 0.21 mm
*V* = 2944.2 (7) Å^3^	

### Data collection

**Table:**

Bruker APEX DUO 4K-CCD diffractometer	14838 independent reflections
Graphite monochromator	13368 reflections with *I* > 2σ(*I*)
Detector resolution: 8.4 pixels mm^-1^	*R*_int_ = 0.053
φ and ω scans	θ_max_ = 28.6°, θ_min_ = 1.2°
Absorption correction: multi-scan SADABS (Bruker, 2008)	*h* = −16→16
*T*_min_ = 0.654, *T*_max_ = 0.850	*k* = −18→18
88599 measured reflections	*l* = −22→22

### Refinement

**Table:**

Refinement on *F*^2^	Primary atom site location: structure-invariant direct methods
Least-squares matrix: full	Secondary atom site location: difference Fourier map
*R*[*F*^2^ > 2σ(*F*^2^)] = 0.070	Hydrogen site location: inferred from neighbouring sites
*wR*(*F*^2^) = 0.181	H-atom parameters constrained
*S* = 1.09	*w* = 1/[σ^2^(*F*_o_^2^) + (0.0475*P*)^2^ + 35.9275*P*] where *P* = (*F*_o_^2^ + 2*F*_c_^2^)/3
14838 reflections	(Δ/σ)_max_ = 0.047
698 parameters	Δρ_max_ = 3.95 e Å^−^^3^
0 restraints	Δρ_min_ = −1.94 e Å^−^^3^

### Special details

**Table:**

Experimental. The intensity data was collected on a Bruker Apex DUO 4 K-CCD diffractometer using an exposure time of 10 s/frame. A total of 3975 frames were collected with a frame width of 0.5° covering up to θ = 28.62° with 98.4% completeness accomplished.
Geometry. All esds (except the esd in the dihedral angle between two l.s. planes) are estimated using the full covariance matrix. The cell esds are taken into account individually in the estimation of esds in distances, angles and torsion angles; correlations between esds in cell parameters are only used when they are defined by crystal symmetry. An approximate (isotropic) treatment of cell esds is used for estimating esds involving l.s. planes.
Refinement. Refinement of F^2^ against ALL reflections. The weighted R-factor wR and goodness of fit S are based on F^2^, conventional R-factors R are based on F, with F set to zero for negative F^2^. The threshold expression of F^2^ > 2sigma(F^2^) is used only for calculating R-factors(gt) etc. and is not relevant to the choice of reflections for refinement. R-factors based on F^2^ are statistically about twice as large as those based on F, and R-factors based on ALL data will be even larger.The aromatic-, methine- and methyl-H atoms were placed in geometrically idealized positions with C—H = 0.95, 1.00, and 0.98 Å, respectively, and allowed to ride on their parent atoms, with *U*_iso_(H) = 1.5*U*_eq_(C) for methyl-H and *U*_iso_(H) = 1.2*U*_eq_(C) for aromatic- and methine-H atoms. Methyl torsion angles were refined from electron density.

### Fractional atomic coordinates and isotropic or equivalent isotropic displacement parameters (Å^2^)

**Table:**

	*x*	*y*	*z*	*U*_iso_*/*U*_eq_	
C1	0.9475 (5)	0.2043 (4)	0.3616 (4)	0.0200 (12)	
C2	0.8727 (5)	0.2354 (4)	0.3038 (4)	0.0182 (11)	
H2	0.8617	0.1993	0.256	0.022*	
C3	0.8146 (5)	0.3197 (4)	0.3169 (4)	0.0223 (13)	
H3	0.7666	0.3407	0.2766	0.027*	
C4	0.8250 (5)	0.3741 (4)	0.3878 (4)	0.0228 (13)	
C5	0.8988 (5)	0.3408 (4)	0.4470 (4)	0.0211 (12)	
H5	0.908	0.376	0.4954	0.025*	
C6	0.9574 (5)	0.2576 (4)	0.4346 (4)	0.0205 (12)	
H6	1.0045	0.2361	0.4752	0.025*	
C7	1.0125 (5)	0.3287 (4)	0.1271 (3)	0.0172 (11)	
C8	1.1043 (5)	0.3261 (4)	0.0816 (4)	0.0222 (12)	
H8	1.1598	0.3723	0.0913	0.027*	
C9	1.1166 (6)	0.2571 (5)	0.0220 (4)	0.0274 (14)	
H9	1.1793	0.2574	−0.0092	0.033*	
C10	1.0370 (6)	0.1878 (5)	0.0083 (4)	0.0271 (14)	
C11	0.9439 (6)	0.1888 (5)	0.0538 (4)	0.0260 (14)	
H11	0.8895	0.1413	0.0451	0.031*	
C12	0.9318 (5)	0.2589 (5)	0.1109 (4)	0.0219 (12)	
H12	0.8674	0.2604	0.1402	0.026*	
C13	0.8761 (5)	0.4836 (4)	0.1781 (4)	0.0186 (11)	
C14	0.8191 (5)	0.4697 (4)	0.1067 (4)	0.0210 (12)	
H14	0.8367	0.418	0.0722	0.025*	
C15	0.7368 (6)	0.5309 (5)	0.0856 (4)	0.0258 (13)	
H15	0.6986	0.5211	0.0368	0.031*	
C16	0.7102 (5)	0.6067 (4)	0.1358 (4)	0.0240 (13)	
C17	0.7668 (5)	0.6216 (4)	0.2071 (4)	0.0238 (13)	
H17	0.7493	0.6736	0.2413	0.029*	
C18	0.8488 (5)	0.5601 (5)	0.2276 (4)	0.0226 (12)	
H18	0.8871	0.5703	0.2763	0.027*	
C19	1.0993 (5)	0.5078 (4)	0.1843 (4)	0.0203 (12)	
C20	1.0782 (5)	0.5693 (5)	0.1217 (4)	0.0232 (13)	
H20	1.0116	0.5632	0.0933	0.028*	
C21	1.1521 (5)	0.6388 (4)	0.1003 (4)	0.0222 (12)	
H21	1.1359	0.6796	0.0579	0.027*	
C22	1.2496 (5)	0.6478 (4)	0.1415 (4)	0.0215 (12)	
C23	1.2724 (5)	0.5878 (5)	0.2036 (4)	0.0230 (13)	
H23	1.3393	0.5941	0.2317	0.028*	
C24	1.1978 (5)	0.5184 (5)	0.2251 (4)	0.0231 (12)	
H24	1.2141	0.478	0.2679	0.028*	
C25	0.7616 (6)	0.4622 (5)	0.4029 (5)	0.0322 (16)	
H25A	0.7462	0.4932	0.3527	0.048*	
H25B	0.8032	0.506	0.438	0.048*	
H25C	0.6936	0.4453	0.4278	0.048*	
C26	1.0125 (5)	0.1142 (4)	0.3513 (4)	0.0207 (12)	
H26	1.0865	0.1269	0.3739	0.025*	
C27	0.9588 (6)	0.0383 (5)	0.4007 (4)	0.0280 (14)	
H27A	0.9638	0.0577	0.4567	0.042*	
H27B	0.9954	−0.0227	0.3927	0.042*	
H27C	0.8828	0.0312	0.3844	0.042*	
C28	1.0233 (6)	0.0813 (5)	0.2656 (4)	0.0278 (14)	
H28A	0.9523	0.0624	0.2437	0.042*	
H28B	1.0719	0.0268	0.2628	0.042*	
H28C	1.0528	0.1336	0.235	0.042*	
C29	1.1271 (9)	0.1161 (7)	−0.0998 (5)	0.049 (2)	
H29A	1.1253	0.1741	−0.1309	0.073*	
H29B	1.1218	0.06	−0.1354	0.073*	
H29C	1.1949	0.1144	−0.0692	0.073*	
C30	0.5940 (7)	0.7367 (5)	0.1615 (6)	0.0390 (19)	
H30A	0.5683	0.7091	0.2103	0.058*	
H30B	0.5354	0.7718	0.1362	0.058*	
H30C	0.6545	0.7804	0.1739	0.058*	
C31	1.3045 (6)	0.7780 (5)	0.0639 (5)	0.0304 (15)	
H31A	1.2944	0.7418	0.014	0.046*	
H31B	1.3646	0.8233	0.0594	0.046*	
H31C	1.2385	0.813	0.0757	0.046*	
C32	0.4449 (5)	0.2814 (4)	0.1359 (4)	0.0190 (12)	
C33	0.3718 (5)	0.2540 (4)	0.1934 (4)	0.0183 (11)	
H33	0.3641	0.292	0.2402	0.022*	
C34	0.3089 (5)	0.1683 (4)	0.1812 (4)	0.0210 (12)	
H34	0.2632	0.148	0.2221	0.025*	
C35	0.3126 (5)	0.1135 (4)	0.1110 (4)	0.0212 (12)	
C36	0.3841 (5)	0.1456 (5)	0.0516 (4)	0.0226 (12)	
H36	0.3881	0.1106	0.003	0.027*	
C37	0.4476 (5)	0.2269 (5)	0.0639 (4)	0.0214 (12)	
H37	0.494	0.2467	0.0233	0.026*	
C38	0.5152 (5)	0.1593 (4)	0.3719 (3)	0.0185 (11)	
C39	0.6095 (6)	0.1654 (5)	0.4173 (4)	0.0250 (13)	
H39	0.6641	0.1195	0.4087	0.03*	
C40	0.6268 (6)	0.2363 (5)	0.4749 (4)	0.0281 (14)	
H40	0.6913	0.2376	0.5062	0.034*	
C41	0.5486 (7)	0.3059 (5)	0.4864 (4)	0.0299 (15)	
C42	0.4540 (6)	0.3013 (5)	0.4414 (4)	0.0280 (14)	
H42	0.4006	0.3485	0.4492	0.034*	
C43	0.4362 (5)	0.2290 (4)	0.3853 (3)	0.0214 (12)	
H43	0.3703	0.2263	0.3557	0.026*	
C44	0.5937 (5)	−0.0197 (4)	0.3173 (4)	0.0189 (11)	
C45	0.5752 (5)	−0.0781 (5)	0.3822 (4)	0.0228 (13)	
H45	0.5114	−0.0699	0.4116	0.027*	
C46	0.6487 (5)	−0.1481 (5)	0.4044 (4)	0.0235 (13)	
H46	0.6348	−0.1869	0.4483	0.028*	
C47	0.7417 (5)	−0.1600 (4)	0.3618 (4)	0.0220 (12)	
C48	0.7613 (5)	−0.1022 (5)	0.2979 (4)	0.0238 (13)	
H48	0.8256	−0.1102	0.2692	0.029*	
C49	0.6883 (5)	−0.0329 (4)	0.2755 (4)	0.0209 (12)	
H49	0.7029	0.0057	0.2316	0.025*	
C50	0.3715 (5)	0.0036 (4)	0.3202 (4)	0.0200 (12)	
C51	0.3168 (5)	0.0175 (5)	0.3909 (4)	0.0221 (12)	
H51	0.3372	0.0691	0.4257	0.027*	
C52	0.2334 (5)	−0.0431 (5)	0.4106 (4)	0.0255 (13)	
H52	0.1972	−0.0331	0.4589	0.031*	
C53	0.2024 (5)	−0.1183 (5)	0.3603 (4)	0.0252 (14)	
C54	0.2562 (5)	−0.1337 (4)	0.2897 (4)	0.0236 (13)	
H54	0.2357	−0.1855	0.2551	0.028*	
C55	0.3400 (5)	−0.0729 (5)	0.2706 (4)	0.0226 (12)	
H55	0.3767	−0.0836	0.2225	0.027*	
C56	0.2434 (6)	0.0267 (5)	0.0959 (5)	0.0296 (15)	
H56A	0.2352	−0.0074	0.1452	0.044*	
H56B	0.2772	−0.0151	0.0566	0.044*	
H56C	0.1723	0.0459	0.0759	0.044*	
C57	0.5140 (6)	0.3707 (4)	0.1460 (4)	0.0242 (13)	
H57	0.5865	0.3565	0.1241	0.029*	
C58	0.4621 (6)	0.4480 (5)	0.0951 (4)	0.0269 (14)	
H58A	0.4603	0.4265	0.0396	0.04*	
H58B	0.5046	0.5072	0.1009	0.04*	
H58C	0.3884	0.4595	0.1126	0.04*	
C59	0.5305 (6)	0.4050 (5)	0.2319 (4)	0.0297 (15)	
H59A	0.4615	0.4258	0.2531	0.045*	
H59B	0.5821	0.4584	0.2344	0.045*	
H59C	0.5585	0.3526	0.2633	0.045*	
C60	0.6452 (9)	0.3841 (7)	0.5933 (6)	0.053 (2)	
H60A	0.6483	0.3251	0.6231	0.079*	
H60B	0.6376	0.4386	0.63	0.079*	
H60C	0.7117	0.3915	0.5637	0.079*	
C61	0.7966 (6)	−0.2890 (5)	0.4415 (5)	0.0300 (15)	
H61A	0.7958	−0.2524	0.4916	0.045*	
H61B	0.8528	−0.3374	0.4442	0.045*	
H61C	0.7263	−0.3202	0.432	0.045*	
C62	0.0789 (7)	−0.2475 (5)	0.3309 (6)	0.041 (2)	
H62A	0.0605	−0.2194	0.28	0.062*	
H62B	0.0144	−0.277	0.3526	0.062*	
H62C	0.1342	−0.2961	0.3233	0.062*	
P1	0.99715 (12)	0.41789 (10)	0.20650 (9)	0.0166 (3)	
P2	0.49275 (13)	0.06929 (10)	0.29344 (9)	0.0169 (3)	
Cl1	1.17183 (12)	0.30968 (11)	0.32444 (10)	0.0246 (3)	
Cl2	1.04584 (14)	0.50981 (11)	0.38312 (9)	0.0242 (3)	
Cl3	0.66582 (12)	0.17348 (11)	0.17376 (10)	0.0254 (3)	
Cl4	0.52593 (13)	−0.02586 (10)	0.11852 (8)	0.0211 (3)	
Ru1	0.98521 (4)	0.35528 (3)	0.33430 (3)	0.01596 (11)	
Ru2	0.47608 (4)	0.13050 (3)	0.16486 (3)	0.01558 (11)	
O1	1.0393 (5)	0.1160 (4)	−0.0478 (3)	0.0378 (13)	
O2	0.6284 (4)	0.6629 (3)	0.1096 (3)	0.0314 (11)	
O3	1.3276 (4)	0.7138 (3)	0.1262 (3)	0.0269 (10)	
O4	0.5567 (6)	0.3797 (4)	0.5406 (3)	0.0430 (14)	
O5	0.8184 (4)	−0.2262 (3)	0.3785 (3)	0.0266 (10)	
O6	0.1193 (4)	−0.1746 (3)	0.3846 (3)	0.0321 (12)	

### Atomic displacement parameters (Å^2^)

**Table:**

	*U*^11^	*U*^22^	*U*^33^	*U*^12^	*U*^13^	*U*^23^
C1	0.022 (3)	0.012 (2)	0.027 (3)	0.000 (2)	0.002 (2)	0.010 (2)
C2	0.018 (3)	0.016 (3)	0.021 (3)	−0.002 (2)	−0.003 (2)	0.004 (2)
C3	0.023 (3)	0.015 (3)	0.029 (3)	−0.002 (2)	0.002 (2)	0.011 (2)
C4	0.021 (3)	0.016 (3)	0.032 (3)	0.001 (2)	0.010 (3)	0.006 (2)
C5	0.026 (3)	0.020 (3)	0.017 (3)	−0.003 (2)	0.005 (2)	0.003 (2)
C6	0.021 (3)	0.021 (3)	0.020 (3)	0.004 (2)	0.003 (2)	0.010 (2)
C7	0.023 (3)	0.012 (2)	0.017 (3)	0.003 (2)	−0.001 (2)	0.0039 (19)
C8	0.023 (3)	0.019 (3)	0.025 (3)	0.002 (2)	0.001 (2)	0.009 (2)
C9	0.035 (4)	0.025 (3)	0.023 (3)	0.004 (3)	0.002 (3)	0.007 (3)
C10	0.041 (4)	0.022 (3)	0.018 (3)	0.005 (3)	−0.002 (3)	0.003 (2)
C11	0.032 (4)	0.022 (3)	0.024 (3)	−0.003 (3)	−0.006 (3)	0.009 (2)
C12	0.023 (3)	0.023 (3)	0.020 (3)	−0.002 (2)	−0.004 (2)	0.008 (2)
C13	0.018 (3)	0.017 (3)	0.020 (3)	−0.002 (2)	0.000 (2)	0.007 (2)
C14	0.023 (3)	0.019 (3)	0.022 (3)	0.000 (2)	−0.001 (2)	0.005 (2)
C15	0.027 (3)	0.026 (3)	0.025 (3)	0.002 (3)	−0.006 (3)	0.006 (3)
C16	0.020 (3)	0.017 (3)	0.036 (4)	−0.003 (2)	−0.004 (3)	0.011 (2)
C17	0.027 (3)	0.015 (3)	0.029 (3)	−0.001 (2)	0.001 (3)	0.003 (2)
C18	0.023 (3)	0.020 (3)	0.024 (3)	−0.001 (2)	−0.003 (2)	0.005 (2)
C19	0.020 (3)	0.020 (3)	0.022 (3)	−0.001 (2)	0.004 (2)	0.005 (2)
C20	0.022 (3)	0.023 (3)	0.025 (3)	−0.004 (2)	−0.003 (2)	0.006 (2)
C21	0.024 (3)	0.018 (3)	0.026 (3)	−0.004 (2)	−0.001 (2)	0.009 (2)
C22	0.022 (3)	0.017 (3)	0.026 (3)	−0.002 (2)	0.001 (2)	0.003 (2)
C23	0.019 (3)	0.020 (3)	0.030 (3)	−0.001 (2)	−0.002 (2)	0.010 (2)
C24	0.024 (3)	0.021 (3)	0.025 (3)	0.000 (2)	−0.001 (2)	0.007 (2)
C25	0.036 (4)	0.019 (3)	0.042 (4)	0.007 (3)	0.014 (3)	0.006 (3)
C26	0.022 (3)	0.017 (3)	0.024 (3)	0.004 (2)	0.000 (2)	0.005 (2)
C27	0.030 (3)	0.019 (3)	0.035 (4)	0.005 (3)	0.002 (3)	0.012 (3)
C28	0.036 (4)	0.019 (3)	0.029 (3)	0.007 (3)	0.005 (3)	0.005 (2)
C29	0.076 (7)	0.038 (4)	0.033 (4)	−0.003 (4)	0.015 (4)	−0.006 (3)
C30	0.031 (4)	0.026 (4)	0.060 (5)	0.007 (3)	−0.015 (4)	0.001 (3)
C31	0.027 (3)	0.021 (3)	0.044 (4)	−0.003 (3)	0.002 (3)	0.014 (3)
C32	0.018 (3)	0.015 (3)	0.025 (3)	0.003 (2)	−0.001 (2)	0.012 (2)
C33	0.020 (3)	0.010 (2)	0.026 (3)	0.007 (2)	0.003 (2)	0.007 (2)
C34	0.021 (3)	0.013 (3)	0.030 (3)	0.000 (2)	0.003 (2)	0.012 (2)
C35	0.021 (3)	0.018 (3)	0.025 (3)	0.000 (2)	−0.004 (2)	0.006 (2)
C36	0.023 (3)	0.025 (3)	0.020 (3)	0.007 (2)	−0.004 (2)	0.002 (2)
C37	0.020 (3)	0.025 (3)	0.020 (3)	0.005 (2)	−0.001 (2)	0.011 (2)
C38	0.023 (3)	0.016 (2)	0.018 (3)	−0.001 (2)	0.003 (2)	0.006 (2)
C39	0.025 (3)	0.027 (3)	0.023 (3)	0.001 (3)	0.001 (2)	0.007 (2)
C40	0.033 (4)	0.027 (3)	0.024 (3)	−0.008 (3)	−0.003 (3)	0.010 (3)
C41	0.049 (4)	0.020 (3)	0.021 (3)	−0.005 (3)	0.007 (3)	0.006 (2)
C42	0.041 (4)	0.018 (3)	0.026 (3)	0.000 (3)	0.009 (3)	0.010 (2)
C43	0.029 (3)	0.021 (3)	0.014 (3)	0.004 (2)	0.002 (2)	0.007 (2)
C44	0.022 (3)	0.017 (3)	0.018 (3)	0.001 (2)	0.000 (2)	0.007 (2)
C45	0.025 (3)	0.021 (3)	0.023 (3)	0.006 (2)	0.005 (2)	0.012 (2)
C46	0.026 (3)	0.020 (3)	0.025 (3)	0.003 (2)	0.003 (2)	0.011 (2)
C47	0.022 (3)	0.017 (3)	0.027 (3)	0.004 (2)	−0.002 (2)	0.004 (2)
C48	0.022 (3)	0.022 (3)	0.028 (3)	0.004 (2)	0.003 (2)	0.004 (2)
C49	0.022 (3)	0.020 (3)	0.021 (3)	0.002 (2)	0.000 (2)	0.008 (2)
C50	0.016 (3)	0.021 (3)	0.023 (3)	0.003 (2)	0.002 (2)	0.010 (2)
C51	0.021 (3)	0.022 (3)	0.024 (3)	0.003 (2)	0.001 (2)	0.008 (2)
C52	0.025 (3)	0.027 (3)	0.026 (3)	0.002 (3)	0.007 (3)	0.011 (3)
C53	0.020 (3)	0.022 (3)	0.035 (4)	0.005 (2)	0.007 (3)	0.015 (3)
C54	0.023 (3)	0.016 (3)	0.032 (3)	0.002 (2)	0.003 (3)	0.006 (2)
C55	0.023 (3)	0.022 (3)	0.024 (3)	0.002 (2)	0.004 (2)	0.006 (2)
C56	0.028 (3)	0.021 (3)	0.039 (4)	0.000 (3)	−0.008 (3)	0.007 (3)
C57	0.025 (3)	0.019 (3)	0.029 (3)	−0.003 (2)	0.000 (3)	0.011 (2)
C58	0.030 (3)	0.017 (3)	0.034 (4)	−0.001 (3)	−0.001 (3)	0.013 (3)
C59	0.038 (4)	0.018 (3)	0.033 (4)	−0.005 (3)	−0.006 (3)	0.007 (3)
C60	0.076 (7)	0.045 (5)	0.038 (5)	−0.003 (5)	−0.008 (5)	0.004 (4)
C61	0.027 (3)	0.022 (3)	0.041 (4)	0.004 (3)	−0.005 (3)	0.014 (3)
C62	0.031 (4)	0.022 (3)	0.072 (6)	−0.006 (3)	0.020 (4)	0.000 (4)
P1	0.0182 (7)	0.0132 (6)	0.0186 (7)	0.0002 (5)	0.0004 (5)	0.0053 (5)
P2	0.0183 (7)	0.0147 (6)	0.0181 (7)	0.0006 (5)	0.0022 (5)	0.0065 (5)
Cl1	0.0194 (7)	0.0231 (7)	0.0319 (8)	0.0034 (6)	0.0028 (6)	0.0115 (6)
Cl2	0.0332 (8)	0.0170 (6)	0.0224 (7)	−0.0044 (6)	0.0021 (6)	0.0003 (5)
Cl3	0.0167 (7)	0.0247 (7)	0.0354 (8)	−0.0018 (6)	−0.0008 (6)	0.0123 (6)
Cl4	0.0266 (7)	0.0166 (6)	0.0203 (6)	0.0057 (5)	0.0009 (5)	0.0028 (5)
Ru1	0.0176 (2)	0.0132 (2)	0.0172 (2)	0.00062 (16)	0.00045 (17)	0.00434 (15)
Ru2	0.0164 (2)	0.0141 (2)	0.0165 (2)	0.00081 (17)	0.00111 (17)	0.00590 (15)
O1	0.058 (4)	0.025 (2)	0.031 (3)	−0.001 (2)	0.005 (3)	0.001 (2)
O2	0.029 (3)	0.020 (2)	0.045 (3)	0.005 (2)	−0.012 (2)	0.009 (2)
O3	0.023 (2)	0.020 (2)	0.039 (3)	−0.0046 (18)	−0.001 (2)	0.011 (2)
O4	0.067 (4)	0.036 (3)	0.025 (3)	−0.007 (3)	0.003 (3)	−0.004 (2)
O5	0.023 (2)	0.022 (2)	0.035 (3)	0.0075 (18)	−0.0001 (19)	0.0100 (19)
O6	0.029 (3)	0.019 (2)	0.049 (3)	−0.0011 (19)	0.014 (2)	0.012 (2)

### Geometric parameters (Å, º)

**Table:**

C1—C2	1.416 (8)	C32—Ru2	2.221 (6)
C1—C6	1.430 (9)	C33—C34	1.434 (8)
C1—C26	1.514 (8)	C33—Ru2	2.222 (6)
C1—Ru1	2.226 (6)	C33—H33	0.95
C2—C3	1.406 (8)	C34—C35	1.402 (9)
C2—Ru1	2.217 (6)	C34—Ru2	2.168 (6)
C2—H2	0.95	C34—H34	0.95
C3—C4	1.408 (10)	C35—C36	1.432 (9)
C3—Ru1	2.182 (7)	C35—C56	1.494 (9)
C3—H3	0.95	C35—Ru2	2.215 (6)
C4—C5	1.434 (9)	C36—C37	1.388 (9)
C4—C25	1.491 (9)	C36—Ru2	2.227 (6)
C4—Ru1	2.217 (6)	C36—H36	0.95
C5—C6	1.395 (9)	C37—Ru2	2.231 (6)
C5—Ru1	2.221 (6)	C37—H37	0.95
C5—H5	0.95	C38—C39	1.387 (9)
C6—Ru1	2.239 (6)	C38—C43	1.410 (9)
C6—H6	0.95	C38—P2	1.825 (6)
C7—C8	1.389 (9)	C39—C40	1.387 (10)
C7—C12	1.411 (9)	C39—H39	0.95
C7—P1	1.827 (6)	C40—C41	1.396 (11)
C8—C9	1.391 (10)	C40—H40	0.95
C8—H8	0.95	C41—O4	1.368 (9)
C9—C10	1.389 (10)	C41—C42	1.386 (11)
C9—H9	0.95	C42—C43	1.384 (9)
C10—O1	1.368 (8)	C42—H42	0.95
C10—C11	1.401 (10)	C43—H43	0.95
C11—C12	1.372 (10)	C44—C49	1.396 (9)
C11—H11	0.95	C44—C45	1.408 (8)
C12—H12	0.95	C44—P2	1.824 (6)
C13—C18	1.393 (9)	C45—C46	1.399 (8)
C13—C14	1.398 (9)	C45—H45	0.95
C13—P1	1.832 (6)	C46—C47	1.383 (9)
C14—C15	1.387 (9)	C46—H46	0.95
C14—H14	0.95	C47—O5	1.367 (7)
C15—C16	1.391 (10)	C47—C48	1.392 (9)
C15—H15	0.95	C48—C49	1.389 (9)
C16—O2	1.366 (8)	C48—H48	0.95
C16—C17	1.393 (10)	C49—H49	0.95
C17—C18	1.386 (9)	C50—C55	1.392 (9)
C17—H17	0.95	C50—C51	1.400 (9)
C18—H18	0.95	C50—P2	1.827 (6)
C19—C24	1.396 (9)	C51—C52	1.384 (9)
C19—C20	1.405 (8)	C51—H51	0.95
C19—P1	1.830 (6)	C52—C53	1.384 (10)
C20—C21	1.390 (8)	C52—H52	0.95
C20—H20	0.95	C53—O6	1.369 (8)
C21—C22	1.387 (9)	C53—C54	1.393 (9)
C21—H21	0.95	C54—C55	1.387 (9)
C22—O3	1.364 (8)	C54—H54	0.95
C22—C23	1.390 (9)	C55—H55	0.95
C23—C24	1.397 (9)	C56—H56A	0.98
C23—H23	0.95	C56—H56B	0.98
C24—H24	0.95	C56—H56C	0.98
C25—H25A	0.98	C57—C59	1.530 (10)
C25—H25B	0.98	C57—C58	1.539 (8)
C25—H25C	0.98	C57—H57	1
C26—C28	1.521 (9)	C58—H58A	0.98
C26—C27	1.526 (8)	C58—H58B	0.98
C26—H26	1	C58—H58C	0.98
C27—H27A	0.98	C59—H59A	0.98
C27—H27B	0.98	C59—H59B	0.98
C27—H27C	0.98	C59—H59C	0.98
C28—H28A	0.98	C60—O4	1.400 (12)
C28—H28B	0.98	C60—H60A	0.98
C28—H28C	0.98	C60—H60B	0.98
C29—O1	1.414 (11)	C60—H60C	0.98
C29—H29A	0.98	C61—O5	1.429 (8)
C29—H29B	0.98	C61—H61A	0.98
C29—H29C	0.98	C61—H61B	0.98
C30—O2	1.417 (10)	C61—H61C	0.98
C30—H30A	0.98	C62—O6	1.431 (10)
C30—H30B	0.98	C62—H62A	0.98
C30—H30C	0.98	C62—H62B	0.98
C31—O3	1.431 (8)	C62—H62C	0.98
C31—H31A	0.98	P1—Ru1	2.3629 (15)
C31—H31B	0.98	P2—Ru2	2.3665 (15)
C31—H31C	0.98	Cl1—Ru1	2.4167 (16)
C32—C33	1.403 (8)	Cl2—Ru1	2.4068 (16)
C32—C37	1.423 (9)	Cl3—Ru2	2.4244 (16)
C32—C57	1.514 (9)	Cl4—Ru2	2.4016 (15)
			
C2—C1—C6	118.3 (5)	O4—C41—C42	115.8 (7)
C2—C1—C26	122.8 (6)	O4—C41—C40	124.8 (7)
C6—C1—C26	118.8 (5)	C42—C41—C40	119.4 (7)
C2—C1—Ru1	71.1 (3)	C43—C42—C41	120.9 (7)
C6—C1—Ru1	71.8 (3)	C43—C42—H42	119.5
C26—C1—Ru1	131.3 (4)	C41—C42—H42	119.5
C3—C2—C1	120.1 (6)	C42—C43—C38	120.4 (6)
C3—C2—Ru1	70.0 (4)	C42—C43—H43	119.8
C1—C2—Ru1	71.7 (3)	C38—C43—H43	119.8
C3—C2—H2	119.9	C49—C44—C45	118.1 (6)
C1—C2—H2	119.9	C49—C44—P2	123.9 (4)
Ru1—C2—H2	131	C45—C44—P2	118.0 (5)
C2—C3—C4	122.2 (6)	C46—C45—C44	121.6 (6)
C2—C3—Ru1	72.7 (4)	C46—C45—H45	119.2
C4—C3—Ru1	72.7 (4)	C44—C45—H45	119.2
C2—C3—H3	118.9	C47—C46—C45	119.3 (6)
C4—C3—H3	118.9	C47—C46—H46	120.4
Ru1—C3—H3	128	C45—C46—H46	120.4
C3—C4—C5	117.4 (6)	O5—C47—C46	123.9 (6)
C3—C4—C25	122.5 (6)	O5—C47—C48	116.3 (6)
C5—C4—C25	120.1 (6)	C46—C47—C48	119.8 (6)
C3—C4—Ru1	70.0 (4)	C49—C48—C47	121.1 (6)
C5—C4—Ru1	71.3 (3)	C49—C48—H48	119.4
C25—C4—Ru1	130.4 (5)	C47—C48—H48	119.4
C6—C5—C4	121.0 (6)	C48—C49—C44	120.2 (6)
C6—C5—Ru1	72.5 (3)	C48—C49—H49	119.9
C4—C5—Ru1	71.0 (3)	C44—C49—H49	119.9
C6—C5—H5	119.5	C55—C50—C51	118.3 (6)
C4—C5—H5	119.5	C55—C50—P2	116.8 (5)
Ru1—C5—H5	129.5	C51—C50—P2	124.5 (5)
C5—C6—C1	120.9 (6)	C52—C51—C50	120.6 (6)
C5—C6—Ru1	71.1 (3)	C52—C51—H51	119.7
C1—C6—Ru1	70.8 (3)	C50—C51—H51	119.7
C5—C6—H6	119.5	C53—C52—C51	120.4 (6)
C1—C6—H6	119.5	C53—C52—H52	119.8
Ru1—C6—H6	131.4	C51—C52—H52	119.8
C8—C7—C12	117.6 (6)	O6—C53—C52	116.4 (6)
C8—C7—P1	121.5 (5)	O6—C53—C54	123.7 (7)
C12—C7—P1	120.9 (5)	C52—C53—C54	119.9 (6)
C7—C8—C9	121.4 (6)	C55—C54—C53	119.4 (6)
C7—C8—H8	119.3	C55—C54—H54	120.3
C9—C8—H8	119.3	C53—C54—H54	120.3
C10—C9—C8	119.9 (7)	C54—C55—C50	121.5 (6)
C10—C9—H9	120	C54—C55—H55	119.3
C8—C9—H9	120	C50—C55—H55	119.3
O1—C10—C9	125.8 (7)	C35—C56—H56A	109.5
O1—C10—C11	114.5 (6)	C35—C56—H56B	109.5
C9—C10—C11	119.7 (6)	H56A—C56—H56B	109.5
C12—C11—C10	119.6 (6)	C35—C56—H56C	109.5
C12—C11—H11	120.2	H56A—C56—H56C	109.5
C10—C11—H11	120.2	H56B—C56—H56C	109.5
C11—C12—C7	121.7 (6)	C32—C57—C59	114.0 (5)
C11—C12—H12	119.1	C32—C57—C58	107.4 (5)
C7—C12—H12	119.1	C59—C57—C58	111.7 (6)
C18—C13—C14	118.7 (6)	C32—C57—H57	107.8
C18—C13—P1	116.4 (5)	C59—C57—H57	107.8
C14—C13—P1	124.5 (5)	C58—C57—H57	107.8
C15—C14—C13	120.5 (6)	C57—C58—H58A	109.5
C15—C14—H14	119.7	C57—C58—H58B	109.5
C13—C14—H14	119.7	H58A—C58—H58B	109.5
C14—C15—C16	120.1 (6)	C57—C58—H58C	109.5
C14—C15—H15	119.9	H58A—C58—H58C	109.5
C16—C15—H15	119.9	H58B—C58—H58C	109.5
O2—C16—C15	115.6 (6)	C57—C59—H59A	109.5
O2—C16—C17	124.5 (6)	C57—C59—H59B	109.5
C15—C16—C17	119.9 (6)	H59A—C59—H59B	109.5
C18—C17—C16	119.5 (6)	C57—C59—H59C	109.5
C18—C17—H17	120.2	H59A—C59—H59C	109.5
C16—C17—H17	120.2	H59B—C59—H59C	109.5
C17—C18—C13	121.2 (6)	O4—C60—H60A	109.5
C17—C18—H18	119.4	O4—C60—H60B	109.5
C13—C18—H18	119.4	H60A—C60—H60B	109.5
C24—C19—C20	117.8 (6)	O4—C60—H60C	109.5
C24—C19—P1	124.0 (5)	H60A—C60—H60C	109.5
C20—C19—P1	118.2 (5)	H60B—C60—H60C	109.5
C21—C20—C19	121.9 (6)	O5—C61—H61A	109.5
C21—C20—H20	119.1	O5—C61—H61B	109.5
C19—C20—H20	119.1	H61A—C61—H61B	109.5
C22—C21—C20	119.3 (6)	O5—C61—H61C	109.5
C22—C21—H21	120.3	H61A—C61—H61C	109.5
C20—C21—H21	120.3	H61B—C61—H61C	109.5
O3—C22—C21	124.5 (6)	O6—C62—H62A	109.5
O3—C22—C23	115.7 (6)	O6—C62—H62B	109.5
C21—C22—C23	119.8 (6)	H62A—C62—H62B	109.5
C22—C23—C24	120.6 (6)	O6—C62—H62C	109.5
C22—C23—H23	119.7	H62A—C62—H62C	109.5
C24—C23—H23	119.7	H62B—C62—H62C	109.5
C19—C24—C23	120.5 (6)	C7—P1—C19	102.9 (3)
C19—C24—H24	119.8	C7—P1—C13	104.5 (3)
C23—C24—H24	119.8	C19—P1—C13	99.0 (3)
C4—C25—H25A	109.5	C7—P1—Ru1	114.85 (18)
C4—C25—H25B	109.5	C19—P1—Ru1	121.1 (2)
H25A—C25—H25B	109.5	C13—P1—Ru1	112.2 (2)
C4—C25—H25C	109.5	C38—P2—C44	102.3 (3)
H25A—C25—H25C	109.5	C38—P2—C50	105.7 (3)
H25B—C25—H25C	109.5	C44—P2—C50	99.2 (3)
C1—C26—C28	113.8 (5)	C38—P2—Ru2	114.78 (19)
C1—C26—C27	106.8 (5)	C44—P2—Ru2	121.3 (2)
C28—C26—C27	112.0 (5)	C50—P2—Ru2	111.5 (2)
C1—C26—H26	108.1	C3—Ru1—C2	37.3 (2)
C28—C26—H26	108.1	C3—Ru1—C4	37.3 (3)
C27—C26—H26	108.1	C2—Ru1—C4	67.5 (2)
C26—C27—H27A	109.5	C3—Ru1—C5	66.9 (2)
C26—C27—H27B	109.5	C2—Ru1—C5	78.9 (2)
H27A—C27—H27B	109.5	C4—Ru1—C5	37.7 (2)
C26—C27—H27C	109.5	C3—Ru1—C1	67.4 (2)
H27A—C27—H27C	109.5	C2—Ru1—C1	37.2 (2)
H27B—C27—H27C	109.5	C4—Ru1—C1	80.4 (2)
C26—C28—H28A	109.5	C5—Ru1—C1	67.1 (2)
C26—C28—H28B	109.5	C3—Ru1—C6	78.6 (2)
H28A—C28—H28B	109.5	C2—Ru1—C6	66.5 (2)
C26—C28—H28C	109.5	C4—Ru1—C6	67.1 (2)
H28A—C28—H28C	109.5	C5—Ru1—C6	36.5 (2)
H28B—C28—H28C	109.5	C1—Ru1—C6	37.4 (2)
O1—C29—H29A	109.5	C3—Ru1—P1	92.34 (17)
O1—C29—H29B	109.5	C2—Ru1—P1	97.58 (16)
H29A—C29—H29B	109.5	C4—Ru1—P1	113.67 (18)
O1—C29—H29C	109.5	C5—Ru1—P1	150.43 (18)
H29A—C29—H29C	109.5	C1—Ru1—P1	125.74 (17)
H29B—C29—H29C	109.5	C6—Ru1—P1	162.96 (17)
O2—C30—H30A	109.5	C3—Ru1—Cl2	122.15 (17)
O2—C30—H30B	109.5	C2—Ru1—Cl2	158.84 (17)
H30A—C30—H30B	109.5	C4—Ru1—Cl2	91.80 (17)
O2—C30—H30C	109.5	C5—Ru1—Cl2	87.36 (17)
H30A—C30—H30C	109.5	C1—Ru1—Cl2	147.33 (17)
H30B—C30—H30C	109.5	C6—Ru1—Cl2	110.54 (17)
O3—C31—H31A	109.5	P1—Ru1—Cl2	86.50 (5)
O3—C31—H31B	109.5	C3—Ru1—Cl1	149.35 (17)
H31A—C31—H31B	109.5	C2—Ru1—Cl1	112.33 (16)
O3—C31—H31C	109.5	C4—Ru1—Cl1	158.28 (18)
H31A—C31—H31C	109.5	C5—Ru1—Cl1	120.70 (18)
H31B—C31—H31C	109.5	C1—Ru1—Cl1	87.69 (17)
C33—C32—C37	118.3 (6)	C6—Ru1—Cl1	92.52 (16)
C33—C32—C57	121.8 (6)	P1—Ru1—Cl1	88.03 (5)
C37—C32—C57	119.7 (5)	Cl2—Ru1—Cl1	88.47 (6)
C33—C32—Ru2	71.6 (3)	C34—Ru2—C35	37.3 (2)
C37—C32—Ru2	71.8 (3)	C34—Ru2—C33	38.1 (2)
C57—C32—Ru2	131.7 (4)	C35—Ru2—C33	68.0 (2)
C32—C33—C34	119.6 (6)	C34—Ru2—C32	67.9 (2)
C32—C33—Ru2	71.5 (3)	C35—Ru2—C32	80.8 (2)
C34—C33—Ru2	68.9 (3)	C33—Ru2—C32	36.8 (2)
C32—C33—H33	120.2	C34—Ru2—C36	66.7 (2)
C34—C33—H33	120.2	C35—Ru2—C36	37.6 (2)
Ru2—C33—H33	132.3	C33—Ru2—C36	78.6 (2)
C35—C34—C33	122.1 (6)	C32—Ru2—C36	67.0 (2)
C35—C34—Ru2	73.2 (4)	C34—Ru2—C37	78.7 (2)
C33—C34—Ru2	73.0 (3)	C35—Ru2—C37	67.1 (2)
C35—C34—H34	119	C33—Ru2—C37	66.0 (2)
C33—C34—H34	119	C32—Ru2—C37	37.3 (2)
Ru2—C34—H34	127	C36—Ru2—C37	36.3 (2)
C34—C35—C36	117.1 (6)	C34—Ru2—P2	92.57 (17)
C34—C35—C56	122.6 (6)	C35—Ru2—P2	113.98 (17)
C36—C35—C56	120.3 (6)	C33—Ru2—P2	98.15 (16)
C34—C35—Ru2	69.5 (4)	C32—Ru2—P2	125.91 (17)
C36—C35—Ru2	71.6 (4)	C36—Ru2—P2	150.73 (18)
C56—C35—Ru2	131.2 (4)	C37—Ru2—P2	163.06 (18)
C37—C36—C35	121.2 (6)	C34—Ru2—Cl4	121.57 (17)
C37—C36—Ru2	72.0 (4)	C35—Ru2—Cl4	91.48 (17)
C35—C36—Ru2	70.8 (4)	C33—Ru2—Cl4	159.07 (17)
C37—C36—H36	119.4	C32—Ru2—Cl4	147.58 (17)
C35—C36—H36	119.4	C36—Ru2—Cl4	87.74 (17)
Ru2—C36—H36	130.5	C37—Ru2—Cl4	110.89 (17)
C36—C37—C32	121.5 (6)	P2—Ru2—Cl4	86.05 (5)
C36—C37—Ru2	71.7 (4)	C34—Ru2—Cl3	149.64 (17)
C32—C37—Ru2	71.0 (3)	C35—Ru2—Cl3	157.78 (17)
C36—C37—H37	119.2	C33—Ru2—Cl3	111.78 (17)
C32—C37—H37	119.2	C32—Ru2—Cl3	87.18 (16)
Ru2—C37—H37	131	C36—Ru2—Cl3	120.25 (18)
C39—C38—C43	117.7 (6)	C37—Ru2—Cl3	92.11 (17)
C39—C38—P2	122.9 (5)	P2—Ru2—Cl3	88.21 (6)
C43—C38—P2	119.3 (5)	Cl4—Ru2—Cl3	88.77 (6)
C40—C39—C38	122.2 (6)	C10—O1—C29	117.0 (6)
C40—C39—H39	118.9	C16—O2—C30	117.1 (6)
C38—C39—H39	118.9	C22—O3—C31	116.5 (5)
C39—C40—C41	119.4 (7)	C41—O4—C60	119.1 (7)
C39—C40—H40	120.3	C47—O5—C61	116.8 (5)
C41—C40—H40	120.3	C53—O6—C62	117.7 (6)
			
C6—C1—C2—C3	3.4 (9)	C6—C5—Ru1—C3	102.4 (4)
C26—C1—C2—C3	179.9 (6)	C4—C5—Ru1—C3	−30.4 (4)
Ru1—C1—C2—C3	−52.6 (5)	C6—C5—Ru1—C2	65.3 (4)
C6—C1—C2—Ru1	56.0 (5)	C4—C5—Ru1—C2	−67.5 (4)
C26—C1—C2—Ru1	−127.5 (6)	C6—C5—Ru1—C4	132.8 (6)
C1—C2—C3—C4	−2.3 (9)	C6—C5—Ru1—C1	28.3 (4)
Ru1—C2—C3—C4	−55.6 (5)	C4—C5—Ru1—C1	−104.5 (4)
C1—C2—C3—Ru1	53.4 (5)	C4—C5—Ru1—C6	−132.8 (6)
C2—C3—C4—C5	0.6 (9)	C6—C5—Ru1—P1	151.0 (3)
Ru1—C3—C4—C5	−55.0 (5)	C4—C5—Ru1—P1	18.2 (6)
C2—C3—C4—C25	−178.5 (6)	C6—C5—Ru1—Cl2	−130.8 (4)
Ru1—C3—C4—C25	125.9 (6)	C4—C5—Ru1—Cl2	96.4 (3)
C2—C3—C4—Ru1	55.7 (5)	C6—C5—Ru1—Cl1	−44.1 (4)
C3—C4—C5—C6	−0.3 (9)	C4—C5—Ru1—Cl1	−177.0 (3)
C25—C4—C5—C6	178.8 (6)	C2—C1—Ru1—C3	28.7 (4)
Ru1—C4—C5—C6	−54.7 (5)	C6—C1—Ru1—C3	−101.1 (4)
C3—C4—C5—Ru1	54.4 (5)	C26—C1—Ru1—C3	146.1 (7)
C25—C4—C5—Ru1	−126.5 (6)	C6—C1—Ru1—C2	−129.8 (5)
C4—C5—C6—C1	1.6 (9)	C26—C1—Ru1—C2	117.5 (7)
Ru1—C5—C6—C1	−52.4 (5)	C2—C1—Ru1—C4	65.2 (4)
C4—C5—C6—Ru1	54.0 (5)	C6—C1—Ru1—C4	−64.6 (4)
C2—C1—C6—C5	−3.1 (9)	C26—C1—Ru1—C4	−177.3 (6)
C26—C1—C6—C5	−179.7 (6)	C2—C1—Ru1—C5	102.1 (4)
Ru1—C1—C6—C5	52.5 (5)	C6—C1—Ru1—C5	−27.7 (4)
C2—C1—C6—Ru1	−55.7 (5)	C26—C1—Ru1—C5	−140.5 (7)
C26—C1—C6—Ru1	127.7 (5)	C2—C1—Ru1—C6	129.8 (5)
C12—C7—C8—C9	0.0 (9)	C26—C1—Ru1—C6	−112.8 (7)
P1—C7—C8—C9	178.9 (5)	C2—C1—Ru1—P1	−47.1 (4)
C7—C8—C9—C10	−1.4 (10)	C6—C1—Ru1—P1	−176.9 (3)
C8—C9—C10—O1	−179.6 (6)	C26—C1—Ru1—P1	70.3 (6)
C8—C9—C10—C11	1.1 (10)	C2—C1—Ru1—Cl2	143.4 (3)
O1—C10—C11—C12	−178.7 (6)	C6—C1—Ru1—Cl2	13.6 (5)
C9—C10—C11—C12	0.6 (10)	C26—C1—Ru1—Cl2	−99.1 (6)
C10—C11—C12—C7	−2.1 (9)	C2—C1—Ru1—Cl1	−133.0 (4)
C8—C7—C12—C11	1.8 (9)	C6—C1—Ru1—Cl1	97.2 (3)
P1—C7—C12—C11	−177.2 (5)	C26—C1—Ru1—Cl1	−15.6 (6)
C18—C13—C14—C15	−0.1 (9)	C5—C6—Ru1—C3	−66.4 (4)
P1—C13—C14—C15	−171.6 (5)	C1—C6—Ru1—C3	67.5 (4)
C13—C14—C15—C16	−0.2 (10)	C5—C6—Ru1—C2	−103.5 (4)
C14—C15—C16—O2	179.8 (6)	C1—C6—Ru1—C2	30.4 (4)
C14—C15—C16—C17	0.6 (10)	C5—C6—Ru1—C4	−29.1 (4)
O2—C16—C17—C18	−179.7 (6)	C1—C6—Ru1—C4	104.8 (4)
C15—C16—C17—C18	−0.6 (10)	C1—C6—Ru1—C5	134.0 (6)
C16—C17—C18—C13	0.3 (10)	C5—C6—Ru1—C1	−134.0 (6)
C14—C13—C18—C17	0.1 (9)	C5—C6—Ru1—P1	−125.4 (5)
P1—C13—C18—C17	172.2 (5)	C1—C6—Ru1—P1	8.6 (8)
C24—C19—C20—C21	0.0 (10)	C5—C6—Ru1—Cl2	53.9 (4)
P1—C19—C20—C21	−179.1 (5)	C1—C6—Ru1—Cl2	−172.2 (3)
C19—C20—C21—C22	0.2 (10)	C5—C6—Ru1—Cl1	143.2 (4)
C20—C21—C22—O3	−179.4 (6)	C1—C6—Ru1—Cl1	−82.9 (3)
C20—C21—C22—C23	−0.2 (10)	C7—P1—Ru1—C3	−80.9 (3)
O3—C22—C23—C24	179.2 (6)	C19—P1—Ru1—C3	154.6 (3)
C21—C22—C23—C24	−0.1 (10)	C13—P1—Ru1—C3	38.2 (3)
C20—C19—C24—C23	−0.3 (10)	C7—P1—Ru1—C2	−43.8 (3)
P1—C19—C24—C23	178.9 (5)	C19—P1—Ru1—C2	−168.4 (3)
C22—C23—C24—C19	0.3 (10)	C13—P1—Ru1—C2	75.3 (3)
C2—C1—C26—C28	21.7 (8)	C7—P1—Ru1—C4	−112.6 (3)
C6—C1—C26—C28	−161.8 (6)	C19—P1—Ru1—C4	122.9 (3)
Ru1—C1—C26—C28	−71.3 (8)	C13—P1—Ru1—C4	6.6 (3)
C2—C1—C26—C27	−102.3 (7)	C7—P1—Ru1—C5	−124.6 (4)
C6—C1—C26—C27	74.2 (7)	C19—P1—Ru1—C5	110.9 (4)
Ru1—C1—C26—C27	164.7 (5)	C13—P1—Ru1—C5	−5.4 (4)
C37—C32—C33—C34	5.1 (8)	C7—P1—Ru1—C1	−17.3 (3)
C57—C32—C33—C34	−179.5 (5)	C19—P1—Ru1—C1	−141.8 (3)
Ru2—C32—C33—C34	−51.3 (5)	C13—P1—Ru1—C1	101.8 (3)
C37—C32—C33—Ru2	56.4 (5)	C7—P1—Ru1—C6	−23.7 (6)
C57—C32—C33—Ru2	−128.2 (5)	C19—P1—Ru1—C6	−148.2 (6)
C32—C33—C34—C35	−4.2 (9)	C13—P1—Ru1—C6	95.4 (6)
Ru2—C33—C34—C35	−56.7 (5)	C7—P1—Ru1—Cl2	157.0 (2)
C32—C33—C34—Ru2	52.4 (5)	C19—P1—Ru1—Cl2	32.5 (2)
C33—C34—C35—C36	1.2 (9)	C13—P1—Ru1—Cl2	−83.8 (2)
Ru2—C34—C35—C36	−55.3 (5)	C7—P1—Ru1—Cl1	68.4 (2)
C33—C34—C35—C56	−176.9 (6)	C19—P1—Ru1—Cl1	−56.1 (2)
Ru2—C34—C35—C56	126.6 (6)	C13—P1—Ru1—Cl1	−172.4 (2)
C33—C34—C35—Ru2	56.6 (5)	C33—C34—Ru2—C35	−132.3 (5)
C34—C35—C36—C37	0.7 (9)	C35—C34—Ru2—C33	132.3 (5)
C56—C35—C36—C37	178.8 (6)	C35—C34—Ru2—C32	104.3 (4)
Ru2—C35—C36—C37	−53.6 (5)	C33—C34—Ru2—C32	−28.0 (3)
C34—C35—C36—Ru2	54.3 (5)	C35—C34—Ru2—C36	30.9 (3)
C56—C35—C36—Ru2	−127.6 (6)	C33—C34—Ru2—C36	−101.4 (4)
C35—C36—C37—C32	0.4 (9)	C35—C34—Ru2—C37	66.9 (4)
Ru2—C36—C37—C32	−52.7 (5)	C33—C34—Ru2—C37	−65.4 (4)
C35—C36—C37—Ru2	53.0 (5)	C35—C34—Ru2—P2	−127.8 (3)
C33—C32—C37—C36	−3.3 (9)	C33—C34—Ru2—P2	99.9 (3)
C57—C32—C37—C36	−178.8 (6)	C35—C34—Ru2—Cl4	−40.8 (4)
Ru2—C32—C37—C36	53.0 (5)	C33—C34—Ru2—Cl4	−173.1 (3)
C33—C32—C37—Ru2	−56.3 (5)	C35—C34—Ru2—Cl3	141.4 (3)
C57—C32—C37—Ru2	128.2 (5)	C33—C34—Ru2—Cl3	9.1 (6)
C43—C38—C39—C40	0.8 (9)	C36—C35—Ru2—C34	129.5 (5)
P2—C38—C39—C40	177.7 (5)	C56—C35—Ru2—C34	−116.0 (8)
C38—C39—C40—C41	−2.0 (10)	C34—C35—Ru2—C33	−29.5 (3)
C39—C40—C41—O4	−179.4 (6)	C36—C35—Ru2—C33	100.0 (4)
C39—C40—C41—C42	1.7 (10)	C56—C35—Ru2—C33	−145.5 (7)
O4—C41—C42—C43	−179.1 (6)	C34—C35—Ru2—C32	−65.5 (4)
C40—C41—C42—C43	−0.1 (9)	C36—C35—Ru2—C32	64.0 (4)
C41—C42—C43—C38	−1.1 (9)	C56—C35—Ru2—C32	178.5 (7)
C39—C38—C43—C42	0.8 (9)	C34—C35—Ru2—C36	−129.5 (5)
P2—C38—C43—C42	−176.2 (5)	C56—C35—Ru2—C36	114.5 (8)
C49—C44—C45—C46	−0.4 (10)	C34—C35—Ru2—C37	−101.8 (4)
P2—C44—C45—C46	−180.0 (5)	C36—C35—Ru2—C37	27.7 (4)
C44—C45—C46—C47	0.1 (11)	C56—C35—Ru2—C37	142.3 (7)
C45—C46—C47—O5	−180.0 (6)	C34—C35—Ru2—P2	59.8 (3)
C45—C46—C47—C48	0.4 (10)	C36—C35—Ru2—P2	−170.7 (3)
O5—C47—C48—C49	179.7 (6)	C56—C35—Ru2—P2	−56.2 (7)
C46—C47—C48—C49	−0.7 (10)	C34—C35—Ru2—Cl4	146.1 (3)
C47—C48—C49—C44	0.4 (10)	C36—C35—Ru2—Cl4	−84.4 (3)
C45—C44—C49—C48	0.2 (10)	C56—C35—Ru2—Cl4	30.2 (6)
P2—C44—C49—C48	179.7 (5)	C34—C35—Ru2—Cl3	−123.5 (4)
C55—C50—C51—C52	−0.2 (9)	C36—C35—Ru2—Cl3	6.0 (7)
P2—C50—C51—C52	−172.1 (5)	C56—C35—Ru2—Cl3	120.5 (6)
C50—C51—C52—C53	−0.4 (10)	C32—C33—Ru2—C34	−133.4 (6)
C51—C52—C53—O6	179.9 (6)	C32—C33—Ru2—C35	−104.5 (4)
C51—C52—C53—C54	0.7 (10)	C34—C33—Ru2—C35	28.9 (4)
O6—C53—C54—C55	−179.6 (6)	C34—C33—Ru2—C32	133.4 (6)
C52—C53—C54—C55	−0.5 (10)	C32—C33—Ru2—C36	−66.7 (4)
C53—C54—C55—C50	−0.1 (10)	C34—C33—Ru2—C36	66.7 (4)
C51—C50—C55—C54	0.5 (9)	C32—C33—Ru2—C37	−30.8 (4)
P2—C50—C55—C54	173.0 (5)	C34—C33—Ru2—C37	102.6 (4)
C33—C32—C57—C59	22.4 (8)	C32—C33—Ru2—P2	142.9 (3)
C37—C32—C57—C59	−162.3 (6)	C34—C33—Ru2—P2	−83.7 (3)
Ru2—C32—C57—C59	−70.9 (8)	C32—C33—Ru2—Cl4	−116.8 (5)
C33—C32—C57—C58	−101.9 (7)	C34—C33—Ru2—Cl4	16.6 (7)
C37—C32—C57—C58	73.4 (7)	C32—C33—Ru2—Cl3	51.5 (4)
Ru2—C32—C57—C58	164.8 (5)	C34—C33—Ru2—Cl3	−175.1 (3)
C8—C7—P1—C19	16.9 (5)	C33—C32—Ru2—C34	28.9 (4)
C12—C7—P1—C19	−164.2 (5)	C37—C32—Ru2—C34	−100.6 (4)
C8—C7—P1—C13	119.9 (5)	C57—C32—Ru2—C34	145.6 (7)
C12—C7—P1—C13	−61.2 (5)	C33—C32—Ru2—C35	65.5 (4)
C8—C7—P1—Ru1	−116.7 (4)	C37—C32—Ru2—C35	−64.1 (4)
C12—C7—P1—Ru1	62.1 (5)	C57—C32—Ru2—C35	−177.9 (6)
C24—C19—P1—C7	−103.7 (6)	C37—C32—Ru2—C33	−129.5 (6)
C20—C19—P1—C7	75.4 (6)	C57—C32—Ru2—C33	116.6 (8)
C24—C19—P1—C13	149.0 (6)	C33—C32—Ru2—C36	102.0 (4)
C20—C19—P1—C13	−31.9 (6)	C37—C32—Ru2—C36	−27.5 (4)
C24—C19—P1—Ru1	26.2 (7)	C57—C32—Ru2—C36	−141.3 (7)
C20—C19—P1—Ru1	−154.7 (4)	C33—C32—Ru2—C37	129.5 (6)
C18—C13—P1—C7	−177.0 (5)	C57—C32—Ru2—C37	−113.9 (7)
C14—C13—P1—C7	−5.3 (6)	C33—C32—Ru2—P2	−47.5 (4)
C18—C13—P1—C19	−71.0 (5)	C37—C32—Ru2—P2	−177.0 (3)
C14—C13—P1—C19	100.7 (6)	C57—C32—Ru2—P2	69.1 (6)
C18—C13—P1—Ru1	58.0 (5)	C33—C32—Ru2—Cl4	143.5 (3)
C14—C13—P1—Ru1	−130.4 (5)	C37—C32—Ru2—Cl4	14.0 (5)
C39—C38—P2—C44	18.3 (6)	C57—C32—Ru2—Cl4	−99.9 (6)
C43—C38—P2—C44	−164.8 (5)	C33—C32—Ru2—Cl3	−133.3 (4)
C39—C38—P2—C50	121.7 (5)	C37—C32—Ru2—Cl3	97.2 (3)
C43—C38—P2—C50	−61.4 (5)	C57—C32—Ru2—Cl3	−16.6 (6)
C39—C38—P2—Ru2	−115.0 (5)	C37—C36—Ru2—C34	103.0 (4)
C43—C38—P2—Ru2	61.8 (5)	C35—C36—Ru2—C34	−30.6 (3)
C49—C44—P2—C38	−105.7 (6)	C37—C36—Ru2—C35	133.6 (6)
C45—C44—P2—C38	73.8 (6)	C37—C36—Ru2—C33	64.9 (4)
C49—C44—P2—C50	145.9 (6)	C35—C36—Ru2—C33	−68.7 (4)
C45—C44—P2—C50	−34.6 (6)	C37—C36—Ru2—C32	28.2 (4)
C49—C44—P2—Ru2	23.7 (7)	C35—C36—Ru2—C32	−105.4 (4)
C45—C44—P2—Ru2	−156.8 (4)	C35—C36—Ru2—C37	−133.6 (6)
C55—C50—P2—C38	−177.2 (5)	C37—C36—Ru2—P2	151.2 (3)
C51—C50—P2—C38	−5.1 (6)	C35—C36—Ru2—P2	17.6 (6)
C55—C50—P2—C44	−71.5 (5)	C37—C36—Ru2—Cl4	−131.0 (4)
C51—C50—P2—C44	100.5 (6)	C35—C36—Ru2—Cl4	95.4 (3)
C55—C50—P2—Ru2	57.5 (5)	C37—C36—Ru2—Cl3	−43.8 (4)
C51—C50—P2—Ru2	−130.5 (5)	C35—C36—Ru2—Cl3	−177.4 (3)
C4—C3—Ru1—C2	133.0 (5)	C36—C37—Ru2—C34	−65.9 (4)
C2—C3—Ru1—C4	−133.0 (5)	C32—C37—Ru2—C34	68.3 (4)
C2—C3—Ru1—C5	−102.3 (4)	C36—C37—Ru2—C35	−28.7 (4)
C4—C3—Ru1—C5	30.7 (4)	C32—C37—Ru2—C35	105.5 (4)
C2—C3—Ru1—C1	−28.6 (3)	C36—C37—Ru2—C33	−103.8 (4)
C4—C3—Ru1—C1	104.4 (4)	C32—C37—Ru2—C33	30.4 (4)
C2—C3—Ru1—C6	−66.0 (4)	C36—C37—Ru2—C32	−134.2 (6)
C4—C3—Ru1—C6	67.0 (4)	C32—C37—Ru2—C36	134.2 (6)
C2—C3—Ru1—P1	99.5 (3)	C36—C37—Ru2—P2	−125.9 (6)
C4—C3—Ru1—P1	−127.6 (3)	C32—C37—Ru2—P2	8.3 (8)
C2—C3—Ru1—Cl2	−173.2 (3)	C36—C37—Ru2—Cl4	53.8 (4)
C4—C3—Ru1—Cl2	−40.2 (4)	C32—C37—Ru2—Cl4	−172.0 (3)
C2—C3—Ru1—Cl1	9.4 (6)	C36—C37—Ru2—Cl3	143.3 (4)
C4—C3—Ru1—Cl1	142.4 (3)	C32—C37—Ru2—Cl3	−82.5 (3)
C1—C2—Ru1—C3	−133.0 (5)	C38—P2—Ru2—C34	−80.6 (3)
C3—C2—Ru1—C4	28.7 (4)	C44—P2—Ru2—C34	155.7 (3)
C1—C2—Ru1—C4	−104.3 (4)	C50—P2—Ru2—C34	39.5 (3)
C3—C2—Ru1—C5	66.4 (4)	C38—P2—Ru2—C35	−112.2 (3)
C1—C2—Ru1—C5	−66.7 (4)	C44—P2—Ru2—C35	124.1 (3)
C3—C2—Ru1—C1	133.0 (5)	C50—P2—Ru2—C35	7.9 (3)
C3—C2—Ru1—C6	102.4 (4)	C38—P2—Ru2—C33	−42.7 (3)
C1—C2—Ru1—C6	−30.6 (4)	C44—P2—Ru2—C33	−166.4 (3)
C3—C2—Ru1—P1	−83.9 (3)	C50—P2—Ru2—C33	77.4 (3)
C1—C2—Ru1—P1	143.1 (3)	C38—P2—Ru2—C32	−16.1 (3)
C3—C2—Ru1—Cl2	16.1 (7)	C44—P2—Ru2—C32	−139.9 (3)
C1—C2—Ru1—Cl2	−116.9 (5)	C50—P2—Ru2—C32	104.0 (3)
C3—C2—Ru1—Cl1	−174.8 (3)	C38—P2—Ru2—C36	−123.8 (4)
C1—C2—Ru1—Cl1	52.1 (4)	C44—P2—Ru2—C36	112.4 (4)
C5—C4—Ru1—C3	129.8 (5)	C50—P2—Ru2—C36	−3.7 (4)
C25—C4—Ru1—C3	−116.1 (8)	C38—P2—Ru2—C37	−22.3 (6)
C3—C4—Ru1—C2	−28.6 (3)	C44—P2—Ru2—C37	−146.1 (6)
C5—C4—Ru1—C2	101.2 (4)	C50—P2—Ru2—C37	97.8 (6)
C25—C4—Ru1—C2	−144.8 (8)	C38—P2—Ru2—Cl4	158.0 (2)
C3—C4—Ru1—C5	−129.8 (5)	C44—P2—Ru2—Cl4	34.2 (2)
C25—C4—Ru1—C5	114.0 (8)	C50—P2—Ru2—Cl4	−81.9 (2)
C3—C4—Ru1—C1	−65.1 (4)	C38—P2—Ru2—Cl3	69.1 (2)
C5—C4—Ru1—C1	64.8 (4)	C44—P2—Ru2—Cl3	−54.7 (2)
C25—C4—Ru1—C1	178.8 (7)	C50—P2—Ru2—Cl3	−170.8 (2)
C3—C4—Ru1—C6	−101.6 (4)	C9—C10—O1—C29	−4.5 (11)
C5—C4—Ru1—C6	28.2 (4)	C11—C10—O1—C29	174.9 (7)
C25—C4—Ru1—C6	142.3 (8)	C15—C16—O2—C30	175.7 (6)
C3—C4—Ru1—P1	59.9 (4)	C17—C16—O2—C30	−5.1 (10)
C5—C4—Ru1—P1	−170.3 (3)	C21—C22—O3—C31	1.0 (10)
C25—C4—Ru1—P1	−56.3 (7)	C23—C22—O3—C31	−178.3 (6)
C3—C4—Ru1—Cl2	146.8 (3)	C42—C41—O4—C60	174.0 (7)
C5—C4—Ru1—Cl2	−83.3 (3)	C40—C41—O4—C60	−4.9 (11)
C25—C4—Ru1—Cl2	30.7 (7)	C46—C47—O5—C61	3.3 (10)
C3—C4—Ru1—Cl1	−122.8 (5)	C48—C47—O5—C61	−177.1 (6)
C5—C4—Ru1—Cl1	7.1 (7)	C52—C53—O6—C62	174.5 (6)
C25—C4—Ru1—Cl1	121.1 (6)	C54—C53—O6—C62	−6.3 (10)

### Hydrogen-bond geometry (Å, º)

*Cg*1 is the centroid of the C1–C6 ring.

**Table:**

*D*—H···*A*	*D*—H	H···*A*	*D*···*A*	*D*—H···*A*
C5—H5···Cl2^i^	0.95	2.62	3.566 (7)	171
C36—H36···Cl4^ii^	0.95	2.6	3.506 (7)	159
C43—H43···Cl1^iii^	0.95	2.78	3.619 (7)	147
C62—H62*B*···O5^iii^	0.98	2.58	3.362 (9)	136
C18—H18···Cl2	0.95	2.8	3.643 (7)	149
C24—H24···Cl1	0.95	2.62	3.427 (6)	143
C49—H49···Cl3	0.95	2.61	3.416 (6)	143
C55—H55···Cl4	0.95	2.71	3.562 (6)	149
C20—H20···*Cg*1	0.95	2.95	3.614 (7)	128

Symmetry codes: (i) −*x*+2, −*y*+1, −*z*+1; (ii) −*x*+1, −*y*, −*z*; (iii) *x*−1, *y*, *z*.
